# Alzheimer’s Disease and Cancer: When Two Monsters Cannot Be Together

**DOI:** 10.3389/fnins.2019.00155

**Published:** 2019-03-01

**Authors:** Shohreh Majd, John Power, Zohreh Majd

**Affiliations:** ^1^Neuronal Injury and Repair Laboratory, Centre for Neuroscience, School of Medicine, Flinders University, Adelaide, SA, Australia; ^2^Psychosomatische Tagesklinik, Passau, Germany

**Keywords:** Alzheimer’s disease, cancer, cell cycle, neurodegeneration, PI3K/Akt/mTOR, beta amyloid, tau phosphorylation, autophagy

## Abstract

Alzheimer’s disease (AD) and cancer are among the leading causes of human death around the world. While neurodegeneration is the main feature of AD, the most important characteristic of malignant tumors is cell proliferation, placing these two diseases in opposite sides of cell division spectrum. Interestingly, AD and cancer’s pathologies consist of a remarkable common feature and that is the presence of active cell cycle in both conditions. In an *in vitro* model of primary adult neuronal culture, we previously showed that treating cell with beta amyloid forced neurons to start a cell cycle. Instead of cell division, however, neuronal cell cycle was aborted and a massive neurodegeneration was left behind as the consequence. A high level of cell cycle entry, which is a requirement for cancer pathogenesis, was reported in clinically diagnosed cases of AD, leading to neurodegeneration. The diverse clinical manifestation of a similar etiology, have puzzled researchers for many years. In fact, the evidence showed an inverse association between AD and cancer prevalence, suggesting that switching pathogenesis toward AD protects patients against cancer and vice versa. In this mini review, we discussed the possibility of involvement of cell proliferation and survival dysregulation as the underlying mechanism of neurodegeneration in AD, and the leading event to develop both disorders’ pathology. As examples, the role of phosphoinositide 3 kinase/Akt/ mammalian target of rapamycin (PI3K/Akt/mTOR) signaling pathway in cell cycle re-entry and blocking autophagy are discussed as potential common intracellular components between AD and cancer pathogenesis, with diverse clinical diagnosis.

## Introduction

Aging is the main risk factor for Alzheimer’s disease (AD) and cancer (White et al., 2014; Guerreiro and Bras, 2015). Although cancer can occur at any age, when it comes to a certain age category, it is not usually seen with AD. Even a history of either of AD or cancer associates with a significant reduced risk of the other, suggesting that these conditions cannot usually meet each other at one time (Romero et al., 2014; Ganguli, 2015; Shi et al., 2015). The main pathological outcome of AD is a massive neurodegeneration and tissue loss throughout the brain, while cancer’s pathology is based on a substantial increase in cell numbers due to an uncontrolled cell division. Understanding how pathogeneses of AD and cancer with a considerable number of common features such as active cell cycle, lead to different outcomes can open the new ways of discovering therapeutic approach for either one or both conditions.

## The Inverse Association Between Alzheimer’s Disease and Cancer

A comprehensive longitudinal study on more than one million participants revealed an inverse association between AD and cancer. In this study, the risk of cancer in the presence of AD was reduced to 50% and the risk of AD in individuals with cancer was decreased by 35% (Musicco et al., 2013). The report was consistent with Roe et al. (2010) findings from another longitudinal study on 6,000 participants over 10 years (1989–1999). Her data demonstrated that a history of cancer decreased the risk of AD, while AD prevalence was also associated with a substantial lower risk of cancer (Roe et al., 2010). Another large 15 years epidemiological study in United States, was further demonstrated a significant reduction in cancer among the patients with AD (Ganguli et al., 2005). Surprisingly, it is not only glioblastoma, the most common form of brain cancer, but other types of cancer such as cancer of lung also reduced the incidence of AD (Sanchez-Valle et al., 2017).

Although the molecular mechanism of this diversity is not clear, the high level of cell cycle activation was found to be a common pathological phenomenon between AD and cancer. Cancer is defined by uncontrolled repeat of cell cycle in an immortal way. On the other side, despite a progressive neurodegeneration in AD brains, the neurons show a substantial increase in their cell cycle related kinases (McShea et al., 2007; Chao et al., 2008; Majd et al., 2008; Moh et al., 2011; D’Angelo et al., 2017). This augmented attempt of neurons to proliferate is believed to start the neurodegenerative events, although its underlying mechanisms is still controversial.

At cellular levels, numerous pathological mechanisms are in common between AD and cancer. An example is involvement of phosphoinositide 3 kinase/Akt/ mammalian target of rapamycin (PI3K/Akt/mTOR) signaling pathway, an essential axis in cell proliferation, metabolism, growth and autophagy in pathogenesis of AD and cancer (Pei and Hugon, 2008; Morgan et al., 2009; Advani, 2010; Talbot et al., 2012; Fumarola et al., 2014; Porta et al., 2014). It is highly possible that the components of this pathway, alone or together, act as one of the common links between AD and cancer, in a same journey of pathogenesis but to different destinations. Identifying those links, might lead us to better therapeutic strategies.

## Original Hypotheses of AD Etiology: Glows and Glooms

For more than a century since Alois Alzheimer has introduced AD for the first time this disease has been recognized by two hallmarks of extracellular senile plaques and intracellular neurofibrillary tangles (NFTs) (Alzheimer, 1906; Majd et al., 2015). Glenner and Wong (1984) reveled the structure of amyloid beta peptide (Aβ) as the main component of senile plaques (Glenner and Wong, 1984). This discovery led to Aβ cascade hypothesis, suggesting Aβ deposition as the first trigger for AD pathogenesis, generating the other hallmarks such as NFTs, neuro-inflammation, synaptic loss and neurodegeneration (Hardy and Selkoe, 2002; Citron, 2004). Finding mutation of amyloid precursor protein (APP) gene on chromosome 21 in many cases of familial AD (FAD) further supported this hypothesis (Goate et al., 1991). Additional investigations demonstrated that in the presence of presenilin 1 (PS1) and presenilin 2 (PS2) mutation, enhanced Aβ production from APP developed the pathology in other types of FAD (Levy-Lahad et al., 1995; Sherrington et al., 1995). Since familial and sporadic AD shared the same pathological hallmarks and clinical manifestation, Aβ hypothesis has been used to explain the pathogenesis in both types of AD.

Beta amyloid hypothesis, however, was challenged when the presence of senile plaques were reported in normal subjects with no clinical symptoms of AD (Dickson et al., 1992). The separate regional localization of Aβ and NFT and v and synaptic loss in AD patients, further questioned the confidence of this hypothesis (Armstrong et al., 1993; Price and Morris, 1999). Meanwhile, other studies suggested a dominant role for tauopathy as initiator of AD pathogenesis. Hyperphosphorylation of tau (p-tau), the main component of NFTs, reduces its glue-like ability in binding actin’s elements to each other to build tubular structure. Instead, p-tau, clumps and builds up NFTs inside the cells (Gomez-Isla et al., 1997; Mitchell et al., 2002; Bennett et al., 2004). Co-presence of NFTs, neurodegeneration and memory impairment in animal (Andorfer et al., 2005; Ramsden et al., 2005) and human studies (Maccioni et al., 2001; Ghoshal et al., 2002) and a significant correlation between p-tau in cerebrospinal fluid (CSF) and the disease progression (Kandimalla et al., 2013), supported tau hypothesis as the other explanation for AD pathology. Tau hypothesis, however, faced the same challenges as Aβ cascade hypothesis. The different spatial and temporal distribution of Aβ plaques and NFTs (Duyckaerts, 2004), along with a considerable level of neuronal death in some brain regions with no NFTs, raised the similar doubt, this time around tau hypothesis as the trigger of events in AD (de la Torre, 2008).

## Cell Cycle Dysregulation Triggers Neurodegeneration

Cell cycle hypothesis has been suggested later as a trigger of AD pathology (Nagy, 2000; Moh et al., 2011). In general, cell cycle divides into four phases of G1 (cell growth), followed by S (DNA synthesis), G2 (cell growth) and eventually M (mitotic) phases. Progressing through different phases is mediated by cellular expression of particular proteins named cyclins and cyclin-dependent kinases (cdks) (Williams and Stoeber, 2012; Barnum and O’Connell, 2014). For example, G1 progression is controlled by cyclin D-cdk4, cyclin D-cdk6, and cyclin E-cdk2 (Planas-Silva and Weinberg, 1997), while cyclin A-cdk2 and cyclin B-cdk1 derive cell progression through G2 and entering into M phase (Nigg, 2001).

Many of these cdks were found elevated in AD neurons, which is an unusual finding for the cells that are considered as post mitotic cells. Although premature neurons experience cell cycle at the beginning of their life in order to differentiate to neurons, they permanently remained in a quiescent state after that for the rest of their life (Lopes et al., 2009). The reason behind this lasting exit from the cell cycle is still a mystery, although the fact that brain tumors have mainly the astrocytes or oligodendrocytes origins but not neuronal origin strongly supports the fact of neuronal inability to divide (Liu and Zong, 2012).

In some circumstances, cell cycle re-entry in adult neurons (from G0 to G1) is followed by cell cycle arrest in an early stage (before G1/S transition point), when re-differentiation is still possible. This transient re-entry into cell cycle in healthy brain might be a part of synaptic remodeling (Arendt, 2003), however, in a large scale, it mainly occurs as a result of insults such as oxidative stress, cytotoxicity or deprivation of neurotrophic factors (Klein and Ackerman, 2003; Kruman, 2004; Chen et al., 2013). Under any of these stress conditions, the control mechanism on G1 to S phase progression fails and cell cycle progression is usually aborted before new DNA synthesis (Liu and Greene, 2001; Becker and Bonni, 2004). Some neurons, however, escape this abortion and enter into S phase, replicate their DNA (Yang et al., 2001; Bonda et al., 2009) and subsequently enter to G2 phase (Nagy et al., 1998; Zhu et al., 2004).

Arriving at G2 phase is the farthest stage that neurons can reach. Why cell division never occurs in mature neurons is still poorly understood, however, a non-canonical pathway of DNA replication, mediated by DNA polymerase β has been suggested as the possible explanation.

It has been shown that degenerative neurons of hippocampal and basal forebrain areas of AD brains have replicated their DNA, either completely or partially. This evidence proved that DNA replication occurs in neuronal cells of affected areas of AD brains, with no similar phenomenon in unaffected regions of the AD brain or in non-demented age-matched subjects (Yang and Herrup, 2007). The cells, however, are largely unable to progress further to complete their division. One likely reason could be the faulty replicated DNA as a result of dominant action of DNA polymerase β in mature neurons.

While in the other cell types DNA polymerase α, γ, or 𝜀 are in charge of DNA replication, in neurons polymerase β partially finalizes this process.

The expression of DNA polymerase β, an error-prone enzyme is frequently over-regulated in AD brains, which is accompanied with the over-expression of proliferating cell nuclear antigen (PCNA) (Zhang et al., 2014). Due to polymerase β error-prone activity replicated DNA contains a few errors. Supercoiling and catenation of duplicated DNA along with the lack of topoisomerase 2, required for successful decatenation and segregation of daughter loops after DNA replication is another reason for inability of neurons to divide (Tiwari et al., 2012; Aranda-Anzaldo and Dent, 2017).

The damaged DNA and unsegregated (catenated) replicons lead the cells to apoptosis (Herrup et al., 2004), however, it is possible that before apoptosis occurs, the neurons remain in a permissive phase of G2 phase for a while. Some studies hypothesize that additional further insults (protein aggregation) is needed for the final progressing of the neurons to death (Yang and Herrup, 2007). This hypothesis was further approved by showing the activation of some cellular mechanisms in G2 phase, deriving the formation of NFTs and Aβ plaques (Baumann et al., 1993; Currais et al., 2009) in apoptosis incompetent neurons. These include a significant increase in APP phosphorylation at Thr^668^ by cdk2, cdk4, and cdk5, which increases its beta-amyloid production and APP proteolysis by the activated caspases during cell cycle (Liu et al., 2003; Chong et al., 2005; Judge et al., 2011; Folch et al., 2012). Overall, abnormal re-entry into the cell cycle is thought to initiate the pathway resulting in NFTs, apoptotic avoidance, and Aβ production (Yang et al., 2003; McShea et al., 2007; Chen et al., 2010).

### Cell Cycle in Cancer

Cell cycle entry is the essential mechanism to develop cancer pathology. Although the majority of the cells either permanently live in a post mitotic status such as neurons or myocytes, or are temporarily withdrawn from it such as glial cells or hepatocytes, a minor group of cells such as epithelial cells are often being into an active phase of proliferation (Williams and Stoeber, 2012). At the checkpoints between cell cycle phases, the transition is tightly regulated besides a close monitoring of genome accuracy (Hartwell and Weinert, 1989; Stumpf et al., 2013; Visconti et al., 2016). Failing to repair DNA damage could end up to apoptosis or persistent genomic abnormality, while repeating this defect in DNA replication leads to cancer pathology, presented by uncontrolled cycles of cell division (Broustas and Lieberman, 2014).

Revealing cell cycle involvement in pathogenesis of both AD and cancer, suggested the same response to some triggers with a possible similar nature. Oxidative stress is widely shown in AD brains (Majd and Power, 2018) and along with metabolic stress, they can be considered as the triggers to initiate cell cycle and developing AD and cancer pathology (Moreira et al., 2010; Sun et al., 2016). The outcome, however, is different in response to a similar trigger. While in AD cell cycle re-entry triggers developing AD hallmarks and neuronal death instead of neuronal proliferation (Liu and Greene, 2001; Becker and Bonni, 2004), in cancer cell death is replaced by unlimited cell division (Broustas and Lieberman, 2014). Given the importance of mitotic mechanisms in AD and cancer, determining the common triggers that stimulate cell cycles’ initiation in both conditions seems critical. Here we discuss the possible involvement of the PI3K/Akt/mTOR pathway as one of the triggers in the pathophysiology of cancer and AD, in relation to cell cycle.

## PI3 Kinase/Akt/mTOR Pathway in Alzheimer’s Disease and Cancer

The PI3K/Akt signaling pathway has a critical regulatory role in cellular growth, proliferation, survival and apoptosis (Yu and Cui, 2016). The main downstream target of PI3K and Akt is mTOR, a peptide with serine/threonine kinase activity. mTOR is a member of PI3K-related kinase (PIKK) family that specifically coordinates cell growth and cell division, by controlling cell cycle progression through different phases (Vadlakonda et al., 2013).

The mTOR peptide is made of two subunits. mTORC1 that is activated by growth factors, nutrients and energy signals, while mTORC2 is less sensitive to energy status and mainly regulates the actin cytoskeleton through its stimulatory impact on PKC-α and Akt (Moschetta et al., 2014). While mTOR constantly monitors oxygen, nutrient and mitogenic signals’ availability (Liu et al., 2009; Zhou and Huang, 2011), its activation is highly regulated by PI3K and Akt (Populo et al., 2012). Interestingly, hyperactivation of PI3K/Akt pathway is often associated with cancer progression (Morgan et al., 2009; Advani, 2010; Fumarola et al., 2014; Porta et al., 2014) and surprisingly contributes to AD pathology during aging (Pei and Hugon, 2008; Talbot et al., 2012).

In animal and cell culture studies dysregulation of PI3K, Akt, and mTOR (Rodon et al., 2013) was led to tumor formation, while knocking out of PI3K, Akt, or mTOR, blocked this oncogenic transformation (Liu et al., 2009), inhibited tumor growth and blocked its invasiveness (Cheng et al., 2005; Slomovitz and Coleman, 2012; Statz et al., 2017). PI3K/Akt/mTOR contribution to AD pathology was demonstrated by numerous evidence (O’Neill, 2013). Activated mTOR is a common finding in AD brains, a possible explanation for cell cycle re-entry in AD (Webber et al., 2005; Bhaskar et al., 2009; Lee et al., 2009; Arendt, 2012; Counts and Mufson, 2017). The role of activated mTOR as a cellular driving force to start proliferation suggests the contribution of mTOR in cell cycle re-entry pathogenesis of AD (Norambuena et al., 2017). mTOR is also believed to increase tau-induced neurodegeneration in a cell cycle-dependent manner (Rogaeva et al., 2006), under the original driving force which could be energy stress as the initiator of abnormal cell cycles (Wen et al., 2004).

## PI3K/Akt/mTOR and Autophagy: Another Common Pathological Mechanism in AD and Cancer

PI3K/Akt/mTOR role in cell cycle initiation is not the only shared pathological mechanism between AD and cancer, underlying neurodegeneration in AD. Activation of mTOR has a negative impact on autophagy, a cytoprotective mechanism to clear toxic proteins such as Aβ. While enhanced autophagy due to mTOR inhibition reduces the aggregation of misfolded proteins and delays neurodegeneration in a wide range of neurodegenerative disorders (Ravikumar et al., 2002; Spencer et al., 2009), enhanced mTOR signaling contributes to inhibition of autophagy, deposition of toxic peptides and progressive neurodegeneration.

Preclinical research evidence supports that mTOR in conjunction with PI3K/Akt pathway regulates autophagy, a mechanism that could assist the body to remove malignant cells in cancer, and clear toxic proteins in AD (Heras-Sandoval et al., 2014). The failure of brain in autophagy and clearing aggregated proteins is one of contributing factors in AD pathology (Bhaskar et al., 2009; Spilman et al., 2010; Li et al., 2013; Pierce et al., 2013). Increase in Aβ deposition (Chen et al., 2009; Cai et al., 2012), decrease its clearance (Pei and Hugon, 2008; Cai et al., 2012), disrupted clearance of tau and the consequent synaptic loss and cognitive decline in AD, all could occur as the result of PI3K/Akt/mTOR activation (Ma et al., 2011; Graziotto et al., 2012; O’Neill, 2013; Yang et al., 2013). A positive impact of mTOR inhibitors in inducing autophagy, increasing Aβ clearance and lessening Aβ aggregation was previously documented (Spilman et al., 2010). Reduced autophagy is also a common pathological finding in cancer (Chang et al., 2012; Cui et al., 2013). Several studies proposed that inhibition of PI3K/Akt/mTOR pathway increased autophagy and effectively reduced the tumor outgrowth (Ji et al., 2015; Butler et al., 2017). Thus, tumorigenesis is promoted when autophagy is compromised.

## A Common Trigger for Neurodegeneration and Cancer

**FIGURE 1:**
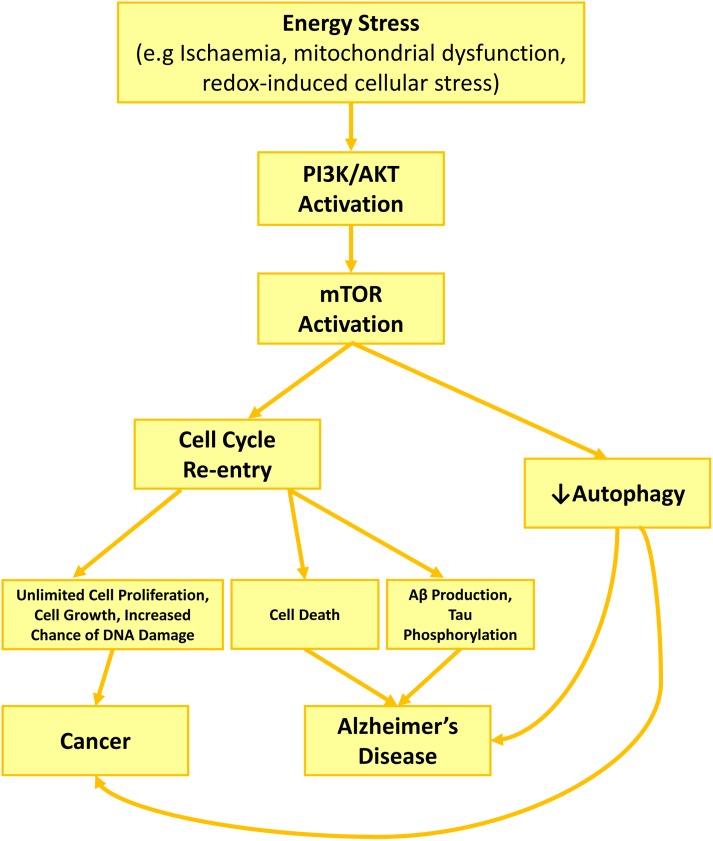
PI3K/Akt/mTOR role in AD development and neurodegeneration through cell cycle activation and autophagy inhibition. Cell stress such as growth factor deprivation or metabolic and oxidative stress enhance PI3K/Akt activity. Akt activation increases Cyclin D1 activity directly or via mTOR activation, while mTOR also activates cdk4. Together enhanced activity of cyclin D1 and cdk4, restart a cell cycle, forcing the neuron to leave G0 and enter G1 phase. Consequent activation of cell cycle kinases, increases APP phosphorylation (cdk2, cdk4, cdk5) toward producing higher amount of Aβ, while activated caspases during cell cycle increase APP proteolysis. Together, increasing in APP phosphorylation and proteolysis lead to higher production and excretion of Aβ, along with high level of phosphorylated tau due to tau kinase activity of cdk2 and cdk5. Activated mTOR also contributes to Aβ accumulation and plaque formation by blocking autophagy and reducing Aβ clearance.

Hyperactivation of PI3K/Akt/mTOR under cell stress conditions could be a mechanism underlying neurodegeneration in AD (Figure 1). Although under cellular stress, PI3K/Akt/mTOR acts as one of the major regulators of cell survival (Datta et al., 1999), its constant activation affect the cells in a negative manner. The repetitive metabolic stress is a shared phenomenon for both AD and cancer (Moreira et al., 2010; Morgen and Frolich, 2015; Sun et al., 2016; Zhao et al., 2017), which could trigger PI3K/Akt/mTOR activation, cell cycle re-entry and autophagy inhibition, the other three shared features between AD and cancer.

Activation of PI3K/Akt/mTOR pathway is a cellular response to metabolic stress in order to promote survival (Yu and Cui, 2016). Phosphorylated PI3K and Akt, during energy stress situation such as mitochondrial stress enhance survival and inhibits apoptosis. To reduce the burden of metabolic pressure on cells, enhanced oxidative phosphorylation occurs through phosphorylation of downstream proteins in an Akt-dependent manner. This strategy to protect the cells against energy stress, however, costs the cells. Activation of Akt inhibits the activity of a few transcription factors such as FoxO that are in charge of regulating the expression of antioxidant enzymes such as superoxide dismutase, while at the same time it activates mTOR (Yang et al., 2017). Consequently, the reduction in antioxidant enzymes expression result in oxidative stress, while the activated mTOR forces the cells into cell cycle and inhibits autophagy. The consequences will be cell cycle progression and autophagy inhibition. Altogether, the activation of cell survival pathway due to energy stress may rescue the cells temporary; however, the long-term consequence could initiate or develop either AD or cancer pathology.

## The Impact of Chronic Stress on AD and Cancer Pathogenesis

A wide range of evidence suggests a strong link between the constant elevation in stress-related hormones including glucocorticoids and epinephrine and development of AD hallmarks such as p-tau and Aβ (Green et al., 2006; Catania et al., 2009; Sotiropoulos et al., 2011; Dioli et al., 2017). It is believed that stress not only associates with misprocessing of APP, leading to Aβ plaques formation, but it interrupts the process of normal autophagy, which results in further buildup of intracellular p-tau and extracellular Aβ accumulation (Silva et al., 2018). Interestingly, chronic stress also associates with a higher risk of cancer development (Cui et al., 2019). Interestingly, PI3K/Akt/mTOR acts as a cross point between AD and cancer, as this pathway is activated under the influence of stress-related hormones, causing cell proliferation and autophagy reduction (Silva et al., 2018; Cui et al., 2019). While PI3K/Akt/mTOR can be considered as a stress-dependent overlapped pathway between AD and cancer, future studies are still required to elucidate the further details, linking stress-related hormones to PI3K/Akt/mTOR dysregulation.

## The Period and Severity of Energy Stress Say the Final Word

While cell stress could be the common trigger for both AD and cancer, the severity of energy stress and the duration that the subject is affected by, seem to be the decision maker in choosing between the two monsters. Compare to AD, which develops within 20 years, cancer could be considered as an acute pathological condition. Uncontrolled cell cycle re-entry in a malignant tissue puts the entire body in a sudden starving situation due to shifting the metabolic resources to affected tissue (Li et al., 2014). Among all the tissues, the brain neurons are the most susceptible cells to suffer from this imbalance between their high metabolic needs and the lack of the proper supply of energy, leading to reactive oxygen species (ROS) accumulation and oxidative damage. This activates a family of FOXO proteins in order to increase the expression of antioxidant enzymes as a protective mechanism to reduce the damage (Hay, 2011; Gomez-Crisostomo et al., 2014). One of the examples is adenosine monophosphate protein kinase protein (AMPK)-dependent FOXO3 activation under metabolic stress (Greer et al., 2007), while mTOR and consequently cell cycle re-entry is also inhibited by AMPK (Xu et al., 2012). Activation of FOXO itself also inhibits mTOR pathway, putting an additional halt on neuronal cell cycle re-entry (Lin et al., 2014). This acute response may override the neuronal PI3K/Akt/mTOR activation (Hay, 2011), with the goal of reducing energy stress and oxidative damage, until the patient either dies or survives from the cancer. In survived patients, this protective response of the brain could provide the subject with a higher level of antioxidant enzymes, less chance for cell cycle re-entry and better protection against AD, at least for a while, compare to normal individuals.

**FIGURE 2:**
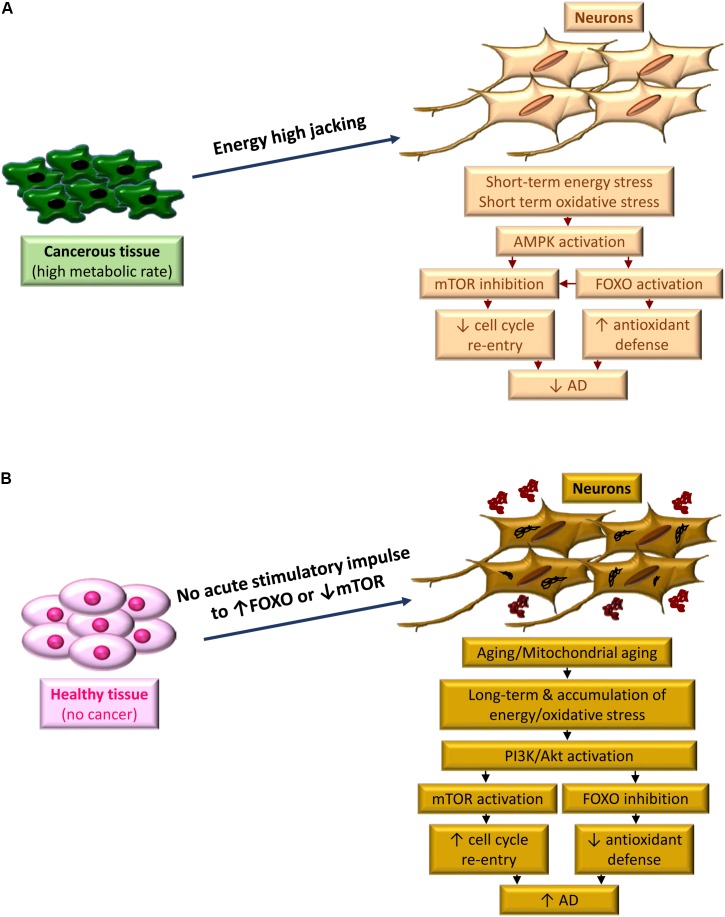
The period of metabolic stress, defines the fate between cancer and AD. **(A)** High metabolic demand of cancerous tissue high jacks energy from highly energy demanded neurons. Acute condition of inadequate supply of energy and concurrent oxidative stress activates AMPK, leading to FOXO activation and m-TOR inhibition. A bust in antioxidant defense occurs along with blocking cell cycle re-entry due to FOXO activation and mTOR inhibition, respectively, which reduces the risk of AD development. **(B)** In the absence of cancer, the acute signal to boost the antioxidant defense of neurons does not exist. Instead, due to aging the neurons, which are facing mitochondrial aging, experience a chronic oxidative stress. This is due to chronic effect of energy stress and accumulation of oxidative damage. Activation of PI3K/Akt pathways dominantly occurs as a protective response, however, this activation leads to further FOXO inhibition and m-TOR activation. Consequently, cell cycle re-entry along with reduced expression of antioxidant enzymes result in progressing of AD pathology.

For the people with AD who show less prevalence of cancer, energy and oxidative stress are not an acute but a chronic phenomenon, associated with mitochondrial aging and accumulated ROS during decades. Depriving from the acute stimulatory effect of cancer on the FOXO machinery and mTOR inhibition in neurons, these cells will gather the accumulated oxidative damage, and higher possibility to restart a cell cycle, within the years. That can lead to the domination of PI3K/Akt/mTOR pathway, Akt-dependent inactivation of FOXO3 (Santo et al., 2013), cell cycle re-entry and AD hallmarks development within a long period of almost 20 years (Figure 2).

## Conclusion

**FIGURE 3:**
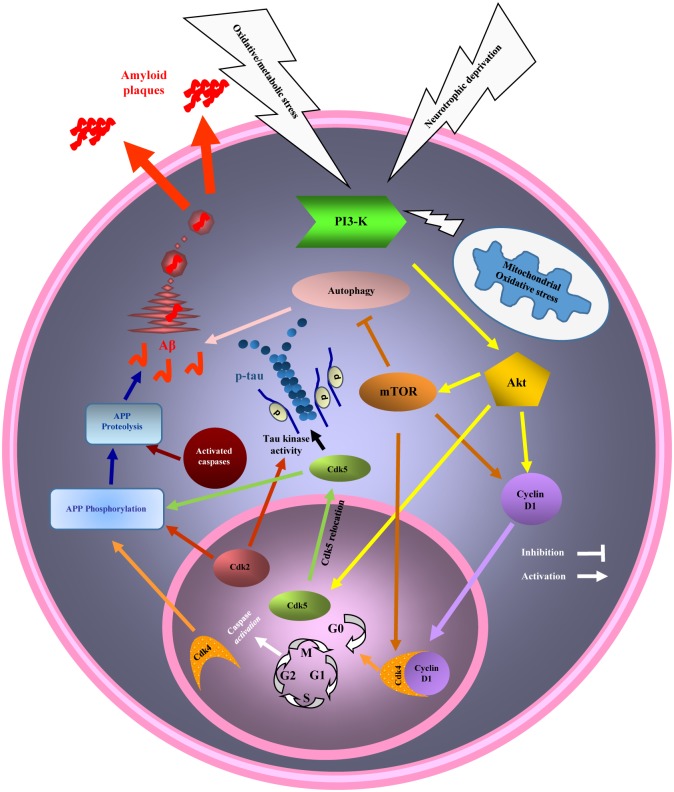
Diagram of the phosphatidylinositol 3 kinase/Akt/mammalian target of the rapamycin (PI3K/Akt/mTOR) pathway and cell cycle and autophagy dysregulation in cancer and Alzheimer’s disease (AD). Mitochondrial stress, a common event in AD and cancer activates PI3K/AKT/mTOR pathway. Activated mTOR, promotes cell cycle re-entry and inhibits autophagy. Cell cycle re-entry contributes in cellular over proliferation, with the higher chance of DNA damage as a result, in cancer. Cell cycle could not be completed by mature neurons and will result in neuronal death (neurodegeneration), and AD hallmarks development (beta amyloid (Aβ) and hyperphosphorylated tau) while the neurons are remained in G2 phase of cell cycle, before death. Inhibition of autophagy due to mTOR activation also participates in AD and cancer pathogenesis.

In conclusion, deregulation of cell cycle as a result of PI3K/Akt/mTOR pathway activation can be considered as a trigger for neurodegeneration in AD and explains the overlapped pathogenesis between AD and cancer with a diverse destiny. The common pathological mechanism that leads to cell growth or survival and cell proliferation in cancer but to a massive cell death in AD could explain inverse association between these diseases (Figure 3). Although many other aspects of a possible explanation for AD neurodegeneration and pathological link connecting AD and cancer are remained to be clarified, cell cycle-related mechanism seems to have a major role in AD and cancer development. Further understanding of this mechanism may open the novel ways to discover therapeutic strategies for either situations or both.

## Author Contributions

All authors were involved in the writing and revising the manuscript.

## Conflict of Interest Statement

The authors declare that the research was conducted in the absence of any commercial or financial relationships that could be construed as a potential conflict of interest.
